# The R.I.R.S. scoring system: An innovative scoring system for predicting stone-free rate following retrograde intrarenal surgery

**DOI:** 10.1186/s12894-017-0297-0

**Published:** 2017-11-21

**Authors:** Yinglong Xiao, Deng Li, Lei Chen, Yaoting Xu, Dingguo Zhang, Yi Shao, Jun Lu

**Affiliations:** 1 Department of Urology, Shanghai General Hospital of Nanjing Medical University, No.100, Haining Road, Hongkou District, Shanghai, 200080 China; 2 Department of Urology, Shanghai Jiao Tong University School of Medicine, Shanghai General Hospital, No.100, Haining Road, Hongkou District, Shanghai, 200080 China; 3 Department of Urology, Branch of Shanghai General Hospital, No. 1878, Middle Sichuan Road, Hongkou District, Shanghai, 200081 China; 4 Department of Urology, Shanghai Pudong New Area People’s Hospital, No. 490, South Chuanhuan road, Shanghai Pudong New Area, Shanghai, 201200 China

**Keywords:** Urolithiasis, Stone surgery, Anatomy, Classification, Prognosis

## Abstract

**Background:**

To establish and internally validate an innovative R.I.R.S. scoring system that allows urologists to preoperatively estimate the stone-free rate (SFR) after retrograde intrarenal surgery (RIRS).

**Methods:**

This study included 382 eligible samples from a total 573 patients who underwent RIRS from January 2014 to December 2016. Four reproducible factors in the R.I.R.S. scoring system, including renal stone density, inferior pole stone, renal infundibular length and stone burden, were measured based on preoperative computed tomography of urography to evaluate the possibility of stone clearance after RIRS.

**Results:**

The median cumulative diameter of the stones was 14 mm, and the interquartile range was 10 to 21. The SFR on postoperative day 1 in the present cohort was 61.5% (235 of 382), and the final SFR after 1 month was 73.6% (281 of 382). We established an innovative scoring system to evaluate SFR after RIRS using four preoperative characteristics. The range of the R.I.R.S. scoring system was 4 to 10. The overall score showed a great significance of stone-free status (*p* < 0.001). The area under the receiver operating characteristic curve of the R.I.R.S. scoring system was 0.904.

**Conclusions:**

The R.I.R.S. scoring system is associated with SFR after RIRS. This innovative scoring system can preoperatively assess treatment success after intrarenal surgery and can be used for preoperative surgical arrangement and comparisons of outcomes among different centers and within a center over time.

## Background

Urolithiasis is one of the most common diseases in urology. It has a prevalence rate of 5.8% in China, of which the most common form is kidney stones [1]. Treatments such as extracorporeal shock wave lithotripsy (SWL), ureteroscopic lithotripsy, retrograde intrarenal surgery (RIRS) and percutaneous nephrolithotomy (PNL) are first-line interventional therapies for urolithiasis according to the European Association of Urology (EAU) guidelines [2]. RIRS, however, has recently become the preferred choice in the management of renal calculi (smaller than 20 mm). Several studies have reported RIRS to be a safe technique, and it is associated with minimal and minor complications for intrarenal stones [3, 4].

As a string of scoring systems have been established for evaluating stone-free rate (SFR) and complications, PNL can be more easily predicted than ever before; [5] yet few criteria remain to preoperatively assess the SFR after RIRS due to a relatively short period of popularization as a first-line treatment for renal stones based on guidelines of the EAU. The Resorlu-Unsal stone score (RUSS) reported by Resorlu et al. can effectively estimate the SFR, and the modified Seoul National University Renal Stone Complexity score (S-ReSC) proposed by Jung et al. can predict the SFR after RIRS on the basis of the affected site without stone size and numbers, [6, 7] however neither of these scoring systems can predict the outcomes of the procedure universally, simply and specifically [8]. In this study, the primary aim was to develop an innovative scoring system and to validate this system with regard to its capacity. This could estimate the SFR preoperatively, minimize complications, and provide a quantification of stone characteristics and patient outcomes between different centers.

## Methods

### R.I.R.S. scoring system

All components of the R.I.R.S. scoring system were obtained by computed tomography urography (CTU), which enabled a confidently reproducible prediction of the stone characteristics [9]. Renal calculus density was defined as an attenuation coefficient of 1 to 2 points as determined by ≤1000 Hu or >1000 Hu. The renal infundibulopelvic angle (RIPA) of the inferior pole stone was defined as the inner angle of the intersection of ureteropelvic axis and the axis of the lower renal calyx. This was separately scored from 1 to 3 points as determined by a non-inferior pole stone or inferior pole stone with RIPA >30° or ≤30°. RIL, the distance from most distal point at bottom stone-containing calix to midpoint of lip of renal pelvis [10] was assigned 2 points and was determined by whether the infundibulopelvic length was more than 25 mm; otherwise, 1 point was recorded. Stone burden, which we described as the cumulative stone diameter (CSD), was obtained by perioperative CTU. We assigned 1 to 3 points for stone burden according to CSD ≤10 mm, >10 mm and ≤20 mm, and >20 mm, respectively. The cumulative stone diameter on CTU was determined using digital calipers (PACS Software Program System) [11].

**Fig. 1:**
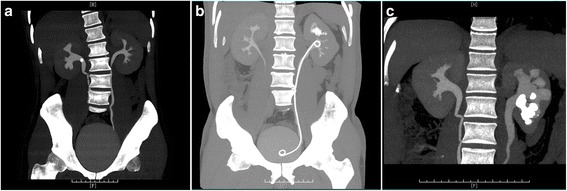
The demonstration of the simplest calculus to the most complicated renal stone presented by computed tomography of urography. **a** The R.I.R.S. score was calculated as 4, the simplest calculus. **b** The R.I.R.S. score was calculated as 7. **c** The R.I.R.S. score was calculated as 10, the most complex situation

A summary of this scoring system ranging from a minimum of 4 to a maximum of 10 points is shown in Table 1. A score of 4 points indicates the simplest calculus, and a score of 10 indicates the most complex situation (Fig. 1).

**Table 1:** Summary of R.I.R.S. scoring system

	Score		
	1	2	3
Renal stone density (Hu)	≤1000	>1000	
Inferior pole stone	non-inferior	inferior with RIPA > 30°	inferior with RIPA ≤ 30°
RIL (mm)	≤25	>25	
Stone burden (mm)	≤10	>10 and ≤20	>20

Range = 4~10 points

### Patients and procedures

All recruited patients underwent RIRS at the Department of Urology in Shanghai General Hospital from January 2014 to December 2016. All participating patients had CTU scans before the surgery to obtain clinical factors. Patients with no available preoperative CTU images, pelviureteric mass, multi-stage procedure and musculoskeletal or renal malformation were excluded. Although the most suitable indication of RIRS is a stone size of less than 20 mm according to the EAU guidelines, several studies have reported favorable results after multi-stage RIRS with lower morbidities [12, 13]. Therefore, we attempted RIRS for larger stones (>20 mm) based on both patient and physician preferences. Postoperative day 1 (POD 1) and postoperative month 1 (POM1) kidney-ureter-bladder (KUB) film were required to estimate surgical outcomes. Additionally, non-contrasted computed tomography (NCCT) was likely to be required if the KUB film at POM1 showed any high-densities, or radiolucent stones in the case of intraoperative findings. A stone-free (SF) status was regarded as no detectable stone on KUB, and fragments of less than 2 mm were also considered negligible stones [14].

Preoperative single-dose antibiotic prophylaxis was used for all patients, however sensitive antibiotics were used for positive urine culture patients until negative [2]. All patients were performed on with a ureteral access sheath (UAS, COOK Medical, USA), which would equally facilitate stone extraction and reduce the intrarenal pressure [15]. A flexible ureteroscope (Olympus type V, Japan) was then advanced through the UAS. The stones were fragmented by holmium: YAG laser lithotripsy, and fragments were removed by a stone extractor (COOK Medical, USA) [16]. If the operation time exceeded 90 min, the procedure was stopped to minimize morbidities. Postoperative double-J stent catheterization was performed and removed at approximately POM1. All procedures were performed under general anesthesia in the lithotomy position by one experienced urologist (Jun Lu).

This study was accepted by the Ethics Review Board of Shanghai General Hospital. All patients were required to provide written informed consent for their data to be used for research purposes.

### Data and statistical analysis

Demographics of the patients, variables of stones and the collecting system, and clinical data were recorded retrospectively. Continuous variables were expressed as the mean ± SD or median (Q3-Q1; interquartile range), and categorical data were presented by *n* (%). The continuous variables were analyzed using Student’s *t* or Mann-Whitney U tests depending on Kruskal-Wallis tests, and categorical data were analyzed by chi-square. The cut-off points of each continuous variable were subjectively set based on Youden’s index, previous literature reviews and clinical practice experience. The multivariate logistic regression models were used to assess the significance of SF status and each score component. Bootstrapping (1000 resamples) was used to internally validate for the R.I.R.S. scoring system. The correlation of the present scoring system with the duration of procedure and hospitalization was tested using correlation analysis. The predictive ability of the R.I.R.S. scoring system was evaluated by the area under the receiver operating characteristic (AUROC) curve. Statistical significance was set at *p* < 0.05. The statistical analysis was performed using SPSS 23.0 (IBM, USA) and presented with GraphPad Prism 6.0 (GraphPad Software, USA).

## Results

Of the 573 patients who underwent RIRS from January 2014 to November 2016, 382 eligible samples were included. The mean renal stone density was 1067.76 ± 336.93 Hu (range 272 to 1899). There were 145 (38.0%) patients who presented with inferior pole stones, and 32 cases were defined as narrow RIPA (≤30°) among those patients. The mean RIL was 23.55 ± 7.53 (range 9 to 63). The median CSD was 14 mm, and the interquartile range was 10 to 21. Other patient demographics and stone characteristics were compared between the SF group and non-SF group and are shown in Table 2 and Table 3.

**Table 2:** Demographics of patients and clinical data

	No. of patients	SF	Non-SF	*P* value
No. of patients (%)	382	281 (73.6%)	101 (26.4%)	
Gender				0.017*
Male	256 (67.0%)	198 (77.3%)	58 (22.7%)	
Female	126 (33.0%)	83 (65.9%)	43 (34.1%)	
Age (yo)	51.93 ± 12.32	52.14 ± 12.19	51.37 ± 12.74	0.590^a^
Hypertention				0.069
Yes	120 (31.4%)	81 (67.5%)	39 (32.5%)	
No	262 (68.6%)	200 (76.3%)	62 (23.7%)	
Diabetes mellitus				0.030*
Yes	36 (9.4%)	21 (57.3%)	15 (41.7%)	
No	346 (90.6%)	260 (75.1%)	86 (24.9%)	
BMI (kg/m2)	21.68 (23.79–19.1; 4.69)	21.57 (23.63–19.13; 4.50)	22.07 (24.29–19.08; 5.21)	0.406^b^
History of surgery				0.064
SWL	13 (3.4%)	11 (84.6%)	2 (15.4%)	
URL or RIRS	36 (9.4%)	22 (61.1%)	14 (38.9%)	
PNL	29 (7.6%)	16 (55.2%)	13 (44.8%)	
Laparoscopic ureterolithotomy	15 (3.9%)	13 (86.7%)	2 (13.3%)	
Pyuloplasty	3 (0.8%)	1 (33.3%)	2 (66.7%)	
Open surgery	3 (0.8%)	2 (66.7%)	1 (33.3%)	
No	294 (77.0%)	223 (75.9%)	71 (24.1%)	
UAS				0.927
Yes	373 (97.6%)	275 (73.7%)	98 (26.3%)	
No	9 (2.4%)	6 (66.7%)	3 (33.3%)	
Relocation				0.317
In situ	346 (90.6%)	252 (72.8%)	94 (27.2%)	
Ex situ	36 (9.4%)	29 (80.6%)	7 (19.4%)	
Duration of procedure (min)	50 (60–40; 20)	50 (60–40; 20)	60 (85–50; 35)	<0.001^b^*
Hospitalization (days)	5 (7–4; 3)	5 (6–3; 3)	6 (7–4; 3)	<0.001^b^*
Complications				0.008*
Yes	27 (7.1%)	14 (51.9%)	13 (48.1%)	
No	335 (92.9%)	267 (75.2%)	88 (24.8%)	

*Statistical significance was set at *P* < 0.05

UAS: Ureteral Access Sheath

^a^Student’s t test, ^b^ Mann-Whitney U test

**Table 3:** Characteristics of stones

	No. of patients	SF	Non-SF	*P* value
No. of patients (%)	382	281 (73.6%)	101 (26.4%)	
Laterality				0.370
Left	201 (52.6%)	144 (71.6%)	57 (28.4%)	
Right	181 (47.4%)	137 (75.7%)	44 (24.3%)	
RIPA (°)	47.91 ± 19.05	52.84 ± 18.51	43.50 ± 18.56	0.003^a^*
Inferior pole stone				<0.001*
Yes	145 (38.0%)	69 (47.6%)	76 (52.4%)	
No	237 (62.0%)	212 (89.5%)	25 (10.5%)	
RIL (mm)	23.55 ± 7.53	22.17 ± 7.02	27.40 ± 7.61	<0.001^a^ *
RIW (mm)	10 (13–8; 5)	10 (12–7; 5)	11 (14–8; 6)	0.036^b^*
Stone burden (mm)	14 (21–10; 11)	12 (17–9; 8)	25 (29–18; 11)	<0.001^b^*
Numbers of stone				<0.001*
Single	233 (61.0%)	200 (85.8%)	33 (32.7%)	
Multiple	149 (39.0%)	81 (54.4%)	68 (45.6%)	
Renal stone density (Hu)	1067.76 ± 336.93	1022.59 ± 342.97	1193.43 ± 285.44	<0.001^a^*
Urine Culture				0.264
Positive	45 (11.8%)	30 (66.7%)	15 (33.3%)	
Negative	337 (88.2%)	251 (74.5%)	86 (25.5%)	

*RIPA* renal infundibulopelvic angle, *RIL* renal infundibulopelvic length, *RIW* renal infundibular width

*Statistical significance was set at *P* < 0.05

^a^Student’s t test, ^b^ Mann-Whitney U test

**Fig. 2:**
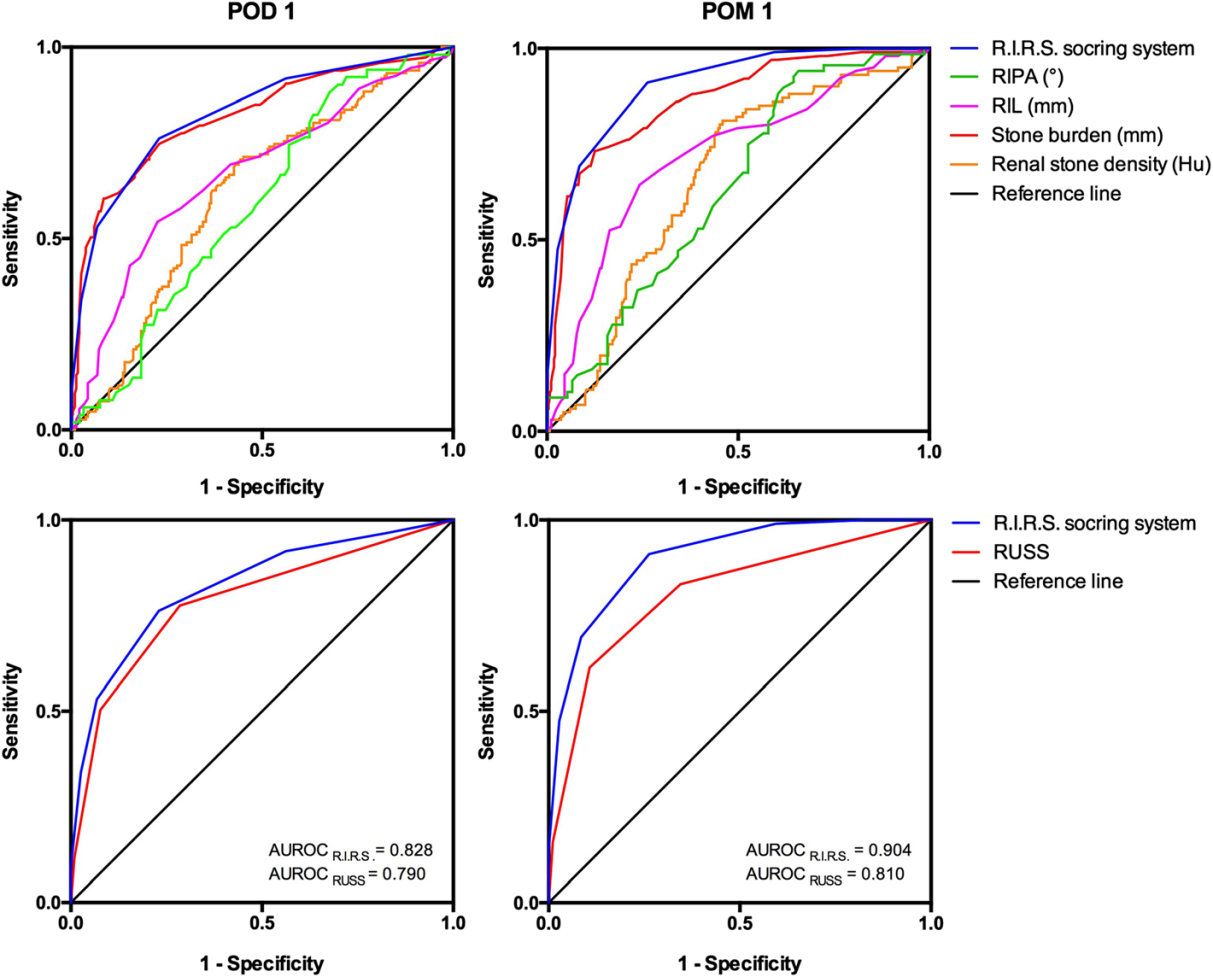
The receiver operating characteristic curves for the R.I.R.S. score compared with individual components and the Resorlu-Unsal stone score

The SFR on POD1 in the present cohort was 61.5% (235 of 382), and the POM1 outcome following RIRS was 73.6% (281 of 382). The SF group and non-SF group were similar with regard to age, hypertension, diabetes, BMI, history of renal surgery, UAS, relocation, laterality and urine culture in univariate analysis; however, gender (*p* = 0.017), diabetes (0 = 0.030), duration of procedure (*p* < 0.001), hospitalization (p < 0.001), complications (*p* = 0.008), RIPA (*p* = 0.003), inferior pole stone (*p* < 0.001), RIL (p < 0.001), renal infundibular width (RIW, *p* = 0.036), renal stone density (*p* < 0.001), numbers of stone (p < 0.001) and stone burden (p < 0.001) showed to be statistically significant. Among these potential variables, the points of renal stone density (*p* = 0.001), inferior pole stone with narrow RIPA (p < 0.001), RIL (*p* = 0.003) and stone burden (*p* < 0.001) were regarded as independent factors in multivariate logistic regression. The R.I.R.S. score of the residual stone group was greater than that of the SF group (8.21 ± 1.22 vs 5.81 ± 1.24). Using bootstrapping (1000 resamples), the R.I.R.S. scoring system correlated with the SF status (*p* < 0.001). Furthermore, a greater R.I.R.S. score was associated with spontaneous clearance (p < 0.001), and correlated with a longer duration of procedure (p < 0.001, correlation coefficient = 0.415). However, the correlation between the R.I.R.S. score and hospitalization did not seem relevant in the present cohort (*p* = 0.009, correlation coefficient = 0.141). The AUROC curve of the R.I.R.S. scoring system for SF status prediction yielded 0.828 in POD1, but 0.904 in POM1 (Fig. 2).

## Discussion

Since the invention of the flexible ureteroscope in the 1970s, advancements in endourological treatments of stones have become increasingly promising. However, because of some limitations, [17] RIRS was not recommended as a first-line treatment for renal calculi until 2013 according to the EAU guidelines. An applicable classification must be established to evaluate RIRS due to its relatively short period of use in clinical practice. In the past few years, a series of scoring systems have been established to estimate the SFR after the procedure, [6, 7, 18] but none of these systems have been effectively and conveniently embraced in clinical practice. Resorlu et al. first reported a scoring system called RUSS to preoperatively classify the probability of SF status [6]. Although RUSS is an uncomplicated and independent predictive scoring system for the SFR, the four-point scoring system might not prognosticate the SFR effectively and comprehensively when considering a complicated scenario in clinical practice [8]. Subsequently, Jung et al. established a modified S-ReSC score, which is based on stone sites without stone burden and numbers [7]. Along with improvements to the technique and experiences, urologists have more choices to manage larger renal stones (≥20 mm), and the stone burden is always considered an indispensable indicator for the SFR [12, 19]. Ito et al. recently performed a nomogram for the SFR after RIRS, but one of the limitations of their study was in the high point range (0–25), which might be time-consuming for physicians [18]. In this study, we aimed to develop and internally validate a novel scoring system for predicting SFR following retrograde intrarenal surgery.

Considering the relation between stone composition and renal calculus density were often reported, meanwhile the maximum renal calculus density measured by Hounsfield Units on CT scans were related to fragmentation efficiency and operative time which would represent stone fragility [20, 21]. We used renal calculus density as the preoperative prediction of outcomes which enabled us to consult with patients, choose the suitable indications and avoid morbidities. We set 1000 Hu of attenuation coefficient as a cut-off point in this study, which was demonstrated to be appropriate for predicting the SF status in a multivariate logistic regression analysis and ROC curve.

Despite the rapid development of a flexible ureteroscope over the past decades, the management of lower pole stones remains a challenge for urologists. Ito et al. retrospectively validated the presence of lower pole calculi and stone size to be independent predictive factors of SF status after RIRS [22, 23]. Subsequently, Jessen et al. reported that a limited RIPA negatively influenced RIRS [24]. We revealed that a narrower RIPA and an increasing stone burden were independent factors in this cohort. These findings support those two variables as critical parameters for predicting outcomes of the procedure.

Inoue et al. retrospectively reviewed that a longer RIL, narrower RIPA and wider RIW would be unpropitious factors. Furthermore, only RIPA <30° was an independent factor for the probability of stone clearance in the multivariate analysis [25]. Resorlu et al. reported that the RIL was longer in the residual stone group compared with the SF group, but this finding was not significant retrospectively [26]. Jessen et al. reported that a negative influence would emerge by a greater RIL for RIRS [24]. In the present study, we discovered that a longer RIL was robustly correlated with SF status in both univariate and multivariate analysis, which was similar to previous literature.

In terms of the reasoning for endoscopic procedures, the stone burden is an independent prognostic factor for the SF status after RIRS, among other characteristics. Ito et al. initially found that the stone area had a relatively lower clinical reliability, while the CSD denoted by KUB films, and, in particular, stone volume described by NCCT is meaningful and impartial predictors of the SF status after RIRS [22]. Considering the inconvenience of calculating stone volume using NCCT by an additional algorithm, the CSD is the most regularly used parameter of stone size in clinical practice [13, 19, 27]. It was further concluded that the CSD obtained would validly and easily estimate the SFR preoperatively [28].

**Fig. 3:**
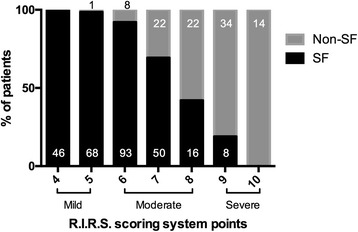
The percentage and number of stone-free and non-stone-free groups stratified by mild, moderate and severe cases

We developed a novel R.I.R.S. scoring system, comprising renal stone density, inferior pole stone, RIL and stone burden. The significance of each factor was defined through statistical analysis to determine the likely SF status after RIRS. Despite nearly overlapping curves with stone burden in POD1, the R.I.R.S. scoring system was found to be strongly prognostic of the final SF status in POM1, which was better than any other variable alone (Fig. 2). These findings supported that, in addition to stone burden, an adverse anatomical condition affects the postoperative spontaneous clearance as well. Moreover, a review of available studies of RIRS demonstrated that operative time was affected by both attenuation coefficients and stone burden, which in turn may affect the complications rate, especially fever and urosepsis [4, 20, 21]. Similar to these findings, the R.I.R.S. score is also correlated with operative time. We believe that it will help urologists to have more appropriate indications of procedure, predict the need for additional sessions of RIRS, and prevent surgical complications. The score was calculated as 4–5 (mild) for 115 (30.1%) patients, 6–8 (moderate) for 211 (55.2%) patients and 9–10 (severe) for 56 (14.7%) patients in this cohort (Fig. 3). The SFR values were 99.1%, 75.4% and 14.3%, respectively. With the intricacy of every procedure and case, the benefits of the R.I.R.S. scoring system appear to be supported by these perioperative observations. All parameters of the scoring system can be easily obtained from regular preoperative testing and do not require any additional software. Moreover, all the variables can provide information about the individual case. To our knowledge, this is the first report of a comprehensive scoring system for predicting SFR after RIRS, which was based on a large cohort of patients. The R.I.R.S. scoring system can favorably predict the outcomes of RIRS, especially the SFR of nephrolithiasis, which could facilitate not only clinical decision making but also patient counseling.

The primary limitations of our study include the retrospective design and analysis of a single center. Another possible study limitation was our exclusion criteria, which included musculoskeletal and renal malformation. These cases were excluded since they were low incidence and do not reflect the typical experience, but affect the outcomes. In addition, since not all patients underwent NCCT for follow-up, the evaluation between preoperative and postoperative imaging may involve a certain degree of bias. Because of these restrictions, the confidence level and bias could not be compared with a prospective and multi-center research study.

## Conclusions

The R.I.R.S. scoring system is associated with SFR after RIRS. This innovative scoring system can be used to preoperatively assess treatment success after intrarenal surgery and can be used for preoperative surgical arrangement and comparison of outcomes among different centers and within a center over time.

## Abbreviations

CSD: Cumulative stone diameter
CTU: Computed tomography of urography
RIL: Renal infundibular length
RIPA: Renal infundibulopelvic length
RIRS: Retrograde intrarenal surgery
RUSS: Resorlu-Unsal stone score
SFR: Stone-free rate
